# Management of Adult Groin Hernia in Denmark: a National Updated Consensus

**DOI:** 10.3389/jaws.2025.14904

**Published:** 2025-08-11

**Authors:** M. Kirk, N. A. Henriksen, M. W. Christoffersen, K. Andresen, A. Dorfelt, M. Krogsgaard, L. Pejtersen, T. Sommer, N. Wensel, N. B. Zinther, F. Helgstrand, J. Rosenberg

**Affiliations:** ^1^ Department of Surgery, Regionshospitalet Viborg, Viborg, Denmark; ^2^ Digestive Disease Center, Bispebjerg Hospital, University of Copenhagen, København, Denmark; ^3^ Department of Surgery, Zealand University Hospital, Koege, Denmark; ^4^ Department of Surgery, Herlev Hospital, University of Copenhagen, København, Denmark; ^5^ Department of Surgery, Odense University Hospital, Odense, Denmark; ^6^ Private Surgical Clinic, Surgical Clinic Svendborg, Svendborg, Denmark; ^7^ Department of Surgery, Moelholm Private Hospital, Vejle, Denmark; ^8^ Department of Surgery, Regionshospitalet Nordjylland, Hjørring, Denmark; ^9^ Department of Surgery, Regionshospitalet Horsens, Horsens, Denmark

**Keywords:** groin hernia, inguinal hernia repair, femoral hernia, laparoscopic surgery, danish hernia database

## Abstract

This article presents the updated Danish national recommendations for the treatment of groin hernias, developed by the Danish Hernia Database steering committee and based on consensus. In alignment with the 2023 European Hernia Society guidelines, a minimally invasive approach is now the preferred strategy for most patients with primary inguinal and femoral hernias, regardless of age or gender. The recommendations emphasize individualized decision-making based on clinical presentation, hernia type, and patient preferences while also integrating national surgical traditions and practices. Key topics include diagnostic criteria, indications for surgery, surgical techniques, management of recurrence, perioperative considerations, and postoperative convalescence. Special attention is given to nerve handling, management of the round ligament in women, and recommendations for both elective and emergency cases. Additionally, the guidelines address changes in surgical education necessitated by the shift toward minimally invasive procedures. The present recommendations apply to Denmark only. These recommendations are adapted to the Danish healthcare environment and may not be directly transferable to other countries, as they reflect local surgical routines, resource availability, and population-specific considerations.

## Introduction

Since the establishment of the Danish Hernia Database in 1998 [1], nationwide data on groin hernia repairs have been collected and reported annually. The unique system, which features personal registration numbers and records for every patient and treatment performed in Denmark, allows for nearly complete data follow-up. Results regarding the quality of surgery are made available to each hospital through a defined set of indicators that outline high-quality treatment. Over the years, data from the database have been utilized for research, contributing to advancements in groin hernia treatment [2].

In 2011, the Danish Hernia Database recommended a treatment algorithm for inguinal and femoral hernias [3]. The goal was to unify treatment at a national level to enhance clinical outcomes for many patients. With the updated guidelines from the European Hernia Society (EHS) in 2023 [4], the treatment algorithm has evolved. There is now a uniform recommendation for a minimally invasive approach to primary groin hernia repair, provided no special circumstances exist. The steering committee of the Danish Hernia Database has developed a new set of national recommendations that take into account special circumstances and traditions while integrating them with the updated international guidelines now available.

This paper aims to adjust the Danish recommendations for treating patients with groin hernias to ensure the continuation and improvement of treatment quality in Denmark. Moreover, with the shift towards an almost entirely minimally invasive approach for the surgical treatment of primary groin hernias, the educational programs for our surgical trainees need to be modified accordingly. The recommendations are summarized in Table 1.

**TABLE 1:** Summary of recommendations.

Diagnosis
Medical history and clinical examination
Consider different diagnosis of no reducible lump is present
Who to treat
Patients seeking treatment for their groin hernia
Patients with a femoral hernia
Patients with bilateral hernias are offered bilateral repair regardless of symptoms from both sides
Method
First choice for primary groin hernias is a minimally invasive mesh repair
In case of recurrence – offer minimally invasive repair after open repair or vice versa
Acute hernias
Reducing the bowel has first priority
Subacute repair if the hernia is reduced
Immediate repair in case of irreducible hernia
TAPP if no fecal peritonitis

### Legal Implications

Practice recommendations are not legal requirements that must be followed in every situation. They reflect best clinical practices based on available evidence and consensus among clinical peers. Since recommendations are developed for the average patient, deviations may be warranted when deemed necessary. In case of deviation from the recommendations, it is advisable to document the reason in the patient’s file.

## Methods

Based on the EHS evidence-based guidelines [4, 5] and the previous recommendations from the Danish Hernia Database [3], we have specifically addressed relevant material for Danish conditions in conjunction with consensus decisions from our national database meetings and several discussions in the steering committee of the Danish Hernia Database. The areas were selected by the steering committee based on their relevance to different regions of the country and their significance to both the public and private healthcare sectors. These recommendations do not fulfil the criteria to be a formal guideline but rely on the available international literature, combined with consensus discussions held at meetings in April 2025. The result is an updated version of our recommendations on the handling of groin hernia in an adult Danish population.

### Diagnosis and Clinical Presentation

In most cases, a clinical examination will reveal a reducible lump in the groin area. When the patient is standing or using their abdominal muscles, the increased intra-abdominal pressure causes the protrusion of fatty tissue and/or bowel into the hernia. The lump in the groin typically disappears when the patient lies down and relaxes. Over time, an increase in size and symptoms may lead to a desire for treatment. Protrusion of the hernia can be accompanied by classic symptoms such as discomfort or pain during rest or physical activity.

Extra care should be taken when making decisions regarding surgery if a reducible lump is absent or if symptoms suggest an etiology other than a groin hernia. Other causes of pain in the groin must be recognized and considered during the examination. Pain in the groin can be caused by reasons other than a groin hernia, such as pain originating from the adductor or iliopsoas muscles, the area around the pubic bone or the hip, or a weakness in the transversalis fascia that leads to pressure on the genitofemoral nerve in the inguinal canal [6].

In cases of doubt, the clinical examination can be supplemented by ultrasound as the primary choice for diagnostic imaging [7]. In experienced hands, ultrasound exhibits higher sensitivity and specificity than other radiological imaging techniques. CT with Valsalva’s maneuver or MRI is available in most hospitals and can be utilized if there is a need to differentiate between other causes of pain in the groin area.

### Indications for Treatment

In general, all patients with symptoms related to a reducible lump in the groin should be offered surgical repair if that is their preference. In the case of a small inguinal hernia without any symptoms, surgical repair should not be routinely recommended, unless it expands in size over time. This strategy is based on data supporting that watchful waiting is a safe approach when no symptoms are present; however, it should be noted that many patients ultimately opt for repair as symptoms develop over time [8].

Femoral hernias require surgical treatment due to the increased risk of incarceration; therefore, every patient with a presumed femoral hernia, regardless of gender, should be offered surgical repair. However, in the rare instance of a femoral hernia without symptoms in a young person, repair may be postponed to later years to minimize the risk of developing chronic pain, provided that the patient is well-informed about the symptoms of strangulation.

In cases of bilateral protruding hernias where only one side causes symptoms, a bilateral minimally invasive repair can be recommended because the asymptomatic side often develops symptoms and may require an additional procedure over time. Patients presenting with a lump indicative of a groin hernia on only one side but with symptoms on both sides should be offered a bilateral repair if a hernia is found on both sides during laparoscopic surgery. An exploration of the groin should involve exposing the area by taking down the peritoneum during laparoscopy.

Diagnostic laparoscopy can be utilized in cases with relevant symptoms but without clinical evidence of a groin hernia. During laparoscopy, the peritoneum should always be incised, and the areas of the internal ring and femoral orifice should be dissected. If a hernia is identified during the procedure, it is repaired laparoscopically. If a lipoma is found in the inguinal canal, it should be reduced, and the area should be covered with a mesh [9]. If no medial, lateral, or femoral hernia is found, and if there is no lipoma in the inguinal canal, the dissection should include visualization of the obturator orifice.

### Preoperative Information

After a detailed medical history, clinical examination, and review of treatment options, the patient should be informed about the surgical plan and its potential complications. Special attention should be given to the risk of persistent groin pain. Generally, the risk of developing severe chronic pain after repair is low. Overall, the occurrence of chronic pain, which can range from minimal to severe, following an open approach is about 11%, while this risk is reduced to around 6% after minimally invasive repair [10], although the evidence behind these numbers is questionable and the true prevalences may be lower [11].

The risk of developing chronic pain increases with younger age, female gender, and high pain scores prior to surgery. As patients age, the risk of chronic pain decreases; however, other factors, such as the risk of incarceration or an enlarged hernia, that may lead to increased surgical complications, should also be considered.

### Surgical Treatment

**FIGURE 1:**
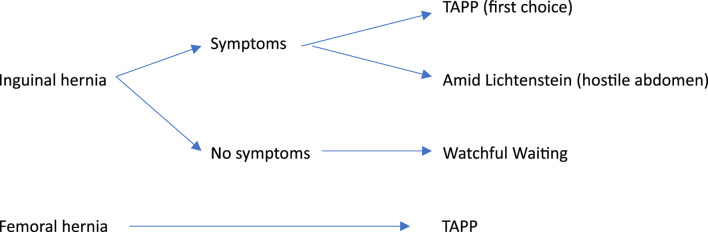
Simplified clinical decision tree for primary groin hernia repair in Denmark.

The preferred surgical treatment for a primary groin hernia is a minimally invasive mesh repair, regardless of the patient’s gender or age (Figure 1). This approach is favored due to its reduced risk of chronic pain, faster recovery, and lower likelihood of surgical site complications [4]. However, certain circumstances might lead to the selection of an open repair instead. This is primarily the case following significant previous surgeries in the pelvic region such as left-sided hemicolectomies, stomas, or the removal of the prostate or bladder, as well as local radiation therapy. If the condition recurs after minimally invasive repair, an Amid Lichtenstein repair is recommended and *vice versa* [5, 12].

In Denmark, routine laparoscopic repair is the transabdominal pre-peritoneal repair (TAPP). While several open techniques exist, the Amid Lichtenstein method is recommended as the standard open tension-free mesh repair [12]. In the elective setting, we recommend that the hernia surgeon can perform the TAPP repair, Amid Lichtenstein repair, Marcy repair, and the modified Lichtenstein repair for the occasional open femoral hernia repair [12].

In the acute setting, reducing hernia content and preventing damage to the bowel takes first priority. A final repair can be performed with or without mesh, depending on the circumstances, or a temporary closure and negative pressure wound therapy may be applied in the contaminated field until a definitive repair can be conducted. Several methods can be utilized, both including minimally invasive repairs such as TAPP or open repairs with or without mesh, such as Amid Lichtenstein, McVay, Bassini or Shouldice for groin hernia or the modified Lichtenstein for a femoral hernia [12]. The preferred method is determined by conditions and local expertise.

For TAPP repair, the mesh should be at least 10 × 15 cm. A medium-weight synthetic mesh is recommended [13]. The mesh can be self-gripping, fixed, or may require no fixation for small lateral hernias. Currently, there is no clear evidence regarding the best method for mesh fixation. The peritoneum is closed with sutures, glue, or tacks, as there is no evidence supporting one method over another. However, in rare cases, penetrating fixation can cause intense and very localized pain; in such cases, early reoperation and removal of the specific tack are recommended. A lightweight synthetic mesh is recommended for open anterior repair [4].

### Classification

To standardize the description of inguinal hernias, the EHS classification [14] should be used in the operative notes. The EHS classification is straightforward, dependable, and comparable, ensuring secure *post hoc* analyses when necessary.

### Operative Technique

For operative techniques, we recommend following the recently published literature [12, 15]. Videos are also available on the Hernia Database website^1^.

### Nerve Handling

During the open Amid Lichtenstein repair, we do not recommend the surgeon actively dissect to locate the three typical nerves in the operative field: the ilioinguinal nerve, the iliohypogastric nerve, and the genital branch of the genitofemoral nerve. If seen, care should be taken not to damage them; therefore, we do not recommend routine nerve division.

During TAPP repair, nerves can be found in the triangle of pain laterally within the operative field. Active dissection in this region to identify the nerves is not recommended, and the placement of penetrating tackers in this area should be avoided.

### Handling of the Round Ligament

In women, the round ligament may complicate mesh placement during laparoscopic repair. In these cases, this structure can be divided to secure adequate mesh placement to prevent recurrences. This is not known to cause long-term complications.

### Recurrence

We recommend using the Amid Lichtenstein procedure if a previous minimally invasive mesh repair has been performed. If a prior open mesh repair has been performed, a minimally invasive approach is recommended. In case of recurrence after both open and minimally invasive repair, a customized approach based on local expertise and patient preference is recommended.

### Antibiotics

The risk of wound or mesh infection is very low, and routine use of antibiotics is not recommended. In high-risk settings, one or more doses may be used as an additional precaution.

### Thrombosis Prophylaxis

Thrombosis prophylaxis is administered after laparoscopic hernia repair. Typically, one dose is given after surgery before discharge. If special circumstances, such as a history of venous thromboembolism, are present, prolonged use may be recommended. Surgeons should be familiar with and adhere to local guidelines regarding the administration of thrombosis prophylaxis. Overall, evidence is limited, and local guidelines should be followed.

### Ambulatory Surgery

Almost all groin hernias should be repaired in an outpatient setting unless specific reasons, such as the need for social support or an unforeseen event, are present.

### Anesthesia and Pain Management

General anesthesia is used for minimally invasive repairs and can also be safely applied in open repair. Local infiltration anesthesia may be utilized for open repairs in patients with significant comorbidities or when there is a local preference. The use of regional anesthesia, such as spinal and epidural anesthesia, is linked to an increased risk of urological complications in patients undergoing inguinal hernia repair [16] and should therefore be avoided.

Opioids for pain management after surgery should be used sparingly, if at all, and are generally unnecessary. If a patient experiences high pain scores immediately following surgery, suspect nerve entrapment or other complications, and consider returning the patient to the operating room for exploration.

If a patient with persistent groin pain returns, medical treatment can be provided at local centers. Both medical treatment and local anesthesia are options for local management. Mapping the pain areas can be beneficial for identifying where to administer local anesthesia in conjunction with an ilioinguinal block. This treatment may be repeated if pain relief occurs. If surgical intervention is needed, treatment has been centralized to a single center in Denmark [17].

### Convalescence and Restrictions

Convalescence recommendations after inguinal hernia repair vary [18], but there is no evidence of an increased risk of recurrence associated with the immediate resumption of normal daily activities. Postoperative pain can significantly contribute to a prolonged recovery; however, most patients should be able to return to work and other daily activities within the first week after surgery. After consensus discussion, however, we recommend waiting on heavy lifting and contact sports for 2–4 weeks after surgery.

### Handling of Irreducible Hernias

Most surgeons will probably prefer an open surgical approach for these patients, but this may change in the coming years. If local expertise is available, laparoscopic repair can be utilized in cases of bowel obstruction caused by an irreducible groin hernia. However, great care should be taken to avoid bowel lesions. When the bowel is repositioned in the abdomen, the hernia can be repaired via TAPP, allowing for easy inspection of the bowel for signs of ischemia. A mesh repair is generally safe, unless there is fecal peritonitis resulting from bowel injury. Fluid from the reaction to reduced fatty tissue or bowel is not a reason to forgo an immediate mesh repair.

In cases of a hernia that can be reduced in the clinic, with or without mild medical relaxation, the hernia can be repaired within the next few days or weeks. When a hernia is reduced, it is advisable to ensure that all the contents of the hernia sac are reduced. If there is uncertainty, a CT scan should be performed if the patient is not taken directly to surgery.

### Training

As minimally invasive repair is the preferred treatment in both elective and acute settings, this type of surgery should be included in the surgical training program for all surgical residents. The Amid Lichtenstein repair will probably not be taught as a routine operation during residency in the near future.

It is important to define the requirements for surgeons caring for elective *versus* acute repairs. In the elective setting, the hernia surgeon should be proficient in TAPP or TEP (TAPP in Denmark) and Amid Lichtenstein. Other techniques, such as the Bassini and the modified Lichtenstein repair, should be known [11]. In the acute setting, all elective repairs can be performed depending on conditions and local expertise. Trainees should be able to perform these repairs under supervision until they are sufficiently skilled in the techniques themselves. Currently, the Shouldice repair is only performed by a few surgeons in Denmark, but if expertise is available, this method can be utilized.

As the number of Amid Lichtenstein repairs rapidly decreases [19], this type of repair is becoming a specialized procedure that should be performed or supervised by a dedicated surgeon to prevent increased recurrence rates.

## Discussion

In this article, we present our updated recommendations on the handling of groin hernia in adults in Denmark. This is based on literature and consensus discussions held at meetings in the Danish Hernia Database. The process was informal and did not involve a vote, as all members of the steering committee reached unanimous agreement on the final recommendations. Since the first publication from the Danish Hernia Database on the handling of groin hernia, many aspects have changed, warranting these updated recommendations.

Laparoscopic surgery is now the preferred treatment for primary groin hernias in both men and women. The open Amid Lichtenstein approach is utilized in cases of recurrence after laparoscopic surgery or when the pelvic region is known to be hostile. We now recommend treating bilateral hernia when found, even if symptoms are only present on one side. In the case of a femoral hernia, surgery is recommended regardless of the patient’s sex or whether symptoms are present.

As the number of open surgeries performed decreases, this procedure should be conducted by dedicated surgeons; therefore, surgical training needs to be adjusted accordingly.

The present recommendations apply to Denmark only, as they were developed in alignment with national surgical practices and healthcare infrastructure. The recommendations are tailored to the Danish healthcare setting and may not be directly transferable to other countries, as they reflect local surgical routines, resource availability, and population-specific considerations.

## Footnotes

1
www.herniedatabasen.dk
